# Evolving serodiagnostics by rationally designed peptide arrays: the *Burkholderia* paradigm in Cystic Fibrosis

**DOI:** 10.1038/srep32873

**Published:** 2016-09-12

**Authors:** Claudio Peri, Alessandro Gori, Paola Gagni, Laura Sola, Daniela Girelli, Samantha Sottotetti, Lisa Cariani, Marcella Chiari, Marina Cretich, Giorgio Colombo

**Affiliations:** 1 Istituto di Chimica del Riconoscimento Molecolare, ICRM, CNR. Via Mario Bianco 9, 20131, Milano (Italy); 2 Cystic Fibrosis Microbiology Laboratory, Fondazione IRCCS Cà Granda, Ospedale Maggiore Policlinico, via San Barnaba 8, 20122, Milano (Italy)

## Abstract

Efficient diagnosis of emerging and novel bacterial infections is fundamental to guide decisions on therapeutic treatments. Here, we engineered a novel rational strategy to design peptide microarray platforms, which combines structural and genomic analyses to predict the binding interfaces between diverse protein antigens and antibodies against *Burkholderia cepacia complex* infections present in the sera of Cystic Fibrosis (CF) patients. The predicted binding interfaces on the antigens are synthesized in the form of isolated peptides and chemically optimized for controlled orientation on the surface. Our platform displays multiple *Burkholderia*-related epitopes and is shown to diagnose infected individuals even in presence of superinfections caused by other prevalent CF pathogens, with limited cost and time requirements. Moreover, our data point out that the specific patterns determined by combined probe responses might provide a characterization of *Burkholderia* infections even at the subtype level (genomovars). The method is general and immediately applicable to other bacteria.

Bacterial infections and epidemics present a continuous threat to mankind through their impact on morbidity and mortality combined to the steady increase of drug-resistance, all factors that make efficient medical intervention strategies and infrastructures urgent necessities. The integration of new research approaches with prevention, care, treatment, and surveillance, can aptly be combined to define the best therapeutic options for affected populations. In this context, the development of quick and effective diagnostic methods is a key factor in patient management, especially for novel, aggressive, or drug-resistant infections that must be tackled in short timeframes. The quest for improved screening and diagnostic approaches includes the development of advanced platforms with the ability to detect and discriminate among the different types of antibodies (IgM, IgG, IgA) from biological fluids, providing fundamental information on infection type and status. State-of-the-art methods entail enzyme immune assays (EIA), commonly employed in the form of ELISA tests, which use antigens (e.g. proteins and lipopolysaccharides) adsorbed on a rigid support as baits to capture antibodies. ELISA is reasonably quick but limited in the number of probes and the use of complete antigens (such as recombinant proteins or complex saccharides) sets severe limitations in terms of cost and versatility^1^,^2^.

The transition to microarray technologies would allow to increase the number of probes and make the tests high-throughput, with the ability to screen simultaneously a large number of molecular probes from the same or multiple pathogens^3^,^4^. Despite the technological advancements represented by protein microarrays, their cost still undermines widespread application in diagnostics as the production of individual recombinant proteins remains a limiting factor.

In this framework, peptide microarrays represent a viable solution to overcome such limitations. Recent initiatives explored the potential of peptide microarrays using libraries of linear peptides spotted on a single chip and interrogated for antigenicity^5^,^6^,^7^. These methods, aimed mainly at epitope mapping, maintain the advantage of a high-throughput test while improving in terms of simplicity and manageability with respect to the use of full-length antigens. In principle, such methodology could be further improved by exploiting highly specific baits, designed as peptide-based mimics that recapitulate the fundamental molecular determinants of antigen recognition^8^,^9^,^10^,^11^. In this context, the rational identification of substructures on selected protein antigens (epitopes) can be translated into the synthesis of easy-to-manage, small sets of *ad hoc* designed peptidic baits displayed on microarrays. The main advantage in this kind of approach stems from the ability to facilitate the analysis and interpretation of results compared to large peptide libraries while exploiting the combination of responses originated from probe redundancy. The use of diverse sets of molecular probes can in fact improve on the reliability of the tests reducing the risk of noise due to unexpected cross-reactivity. Furthermore, the analysis of combined signal patterns opens new perspectives for serologic diagnosis: the presence and the combined response of antibodies directed against their specific synthetic epitopes could report on the status of the infection, identify patient subgroups, discriminate different pathogens (and their variants) and assist in medical decision making. Peptide-focused diagnostic design is a conceptually fascinating but still highly unexplored avenue.

Here, we present an original integration of the results of computational epitope design, peptide synthesis and optimal modification for probe display on microarrays with the aim to move the application of molecular diagnostics beyond its current limits: in the case of a highly invalidating rare disease such as Cystic Fibrosis (CF), we prove the possibility to generate highly efficient, selective peptide-based microarray diagnostic tools that can be predictive of the infection state as well as show potential for the definition of the genomic variant of the pathogen. Specifically, we describe a novel platform for the screening of *Burkholderia cepacia* complex (BCC) infections in subjects affected by Cystic Fibrosis (CF).

Chronic and recurrent bacterial infections often characterize lung conditions in CF. Even if advances in antibiotic therapy contribute enormously to increase survival in CF, the growth of bacterial biofilms in CF airways can make their eradication extremely difficult – even aggressive antibiotic treatments can be ineffective when facing multidrug-resistant organisms. Routine laboratory techniques can identify only a small fraction of the microbes present in the CF airway^12^ and currently there is no readily available methodology to discriminate among these organisms to guide clinical therapeutic decisions^13^. In this scenario, the most prevalent pathogen *P*. *aeruginosa*, and others such as *Achromobacter xylosoxidans* and the *Burkholderia cepacia* complex often resist treatment, leading to respiratory failure, with lung transplantation being the only therapeutic option^14^. *Burkholderia cepacia* complex (BCC) bacteria have gained notoriety in CF due to the difficulties they pose in diagnosis and treatment. They are associated to a poor prognosis and can also easily spread amongst CF individuals. This group of closely related *Burkholderia* species can be further classified into 17 different genomovars^15^. Rapid and accurate diagnosis is the key to select the best therapeutic strategy and to control such infections. However, BCC diagnosis is currently based on bacterial culture isolation and sequencing, which is a cumbersome and time consuming process requiring specialized laboratories^16^ while ELISA tests for antibody detection are generally unstandardized and so far not widely used in clinics due to cross-reactivity issues.

The selective detection of *BCC* species in CF patients, even in the face of other common respiratory superinfections, represent thus an optimal benchmark for our rationally-designed peptide microarray platform to verify its ability at providing a test that is fast, effective, reliable and specific. A visual overview of the methodology employed is summarized in Fig. 1.

Very briefly, the platform proved to be highly predictive, with seven probes discriminating correctly the BCC positive samples with confidence exceeding 97%, and differences in signal distributions relative to the control characterized by good statistical significance (p < 0.001).

Since it starts from the knowledge and re-design of conserved epitopes, our method is fully general and applicable to any pathogen for which structural information on antigenic proteins is available and it may be immediately extended to the screening of multiple infections on a single chip.

## Results

### Selection of the probes

**Figure 2:**
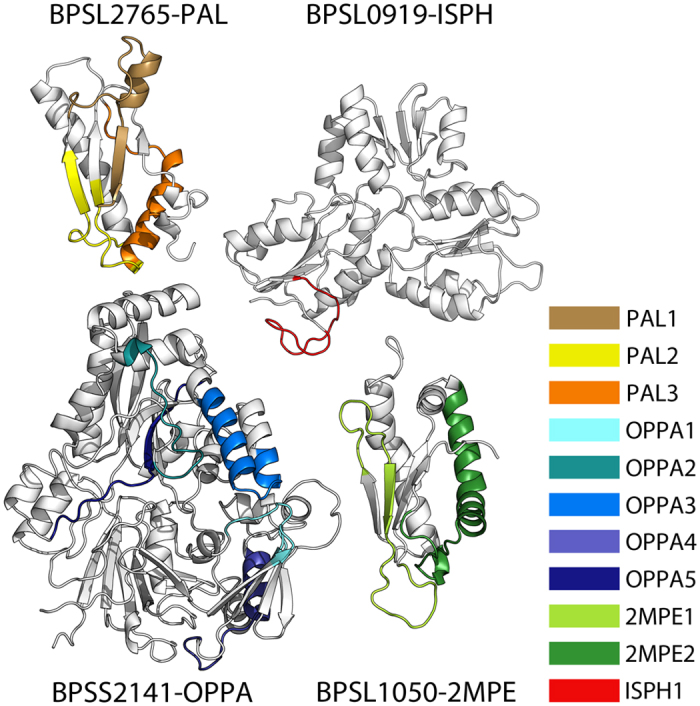
Visual representation of the epitopes in their native structural context. The figure shows all four antigens from *B*. *pseudomallei* chosen for this study and their corresponding epitopes highlighted in different colors. The PDB codes used for each antigen are 4B5C, 3ZS6 and 2MPE for proteins BPSL2765, BPSS2141 and BPSL1050 respectively. The structure of BPSL0919 was reconstructed by homology modeling from template 3F7T.

Each of the selected peptidic probes, corresponding to either a linear or conformational immunoreactive epitope, was rationally designed on the basis of the three-dimensional structure of cognate antigenic protein, as described in previous papers^8^,^9^,^10^,^11^. The list of antigens and corresponding epitopes is described in Table 1 and shown as Fig. 2.

Specifically, to establish the suitability of designed epitopes for BCC applications in CF, we performed a sequence conservation analysis of the selected epitopes against six genomovars of the *cepacia* complex (*B*. *cepacia*, *B*. *multivorans*, *B*.*cenocepacia*, *B*. *vietnamiensis*, *B*. *dolosa*, *B ambifaria*) using the EnseblGenomes resources (http://ensemblgenomes.org)^17^. The analysis also included other relevant *Burkholderia* species (*B*. *mallei*, pathogenic, and *B*. *thailandensis*, usually non-pathogenic) and common, but unrelated, pathogenic microbes affecting CF patients (*P*. *aeruginosa*, *A*. *xylosoxidans*, *S*. *aureus*, *S*. *maltophilia*) and a fungus, *C*. *albicans* (Supplementary Figure S1). The results confirmed that the selected epitopes are well conserved and mostly identical among the different *Burkholderia* species, while poorly conserved if not totally absent in all other pathogens, with the sole exception of epitope ISPH1 from protein BPSL0919 (Supplementary Figure S1). The latter antigen appears to be homologue to other proteins from the same family across the *Burkholderia* genus, *P*. *aeruginosa* and *A*. *xylosoxidans*.

In previous reports, all epitopes except for ISPH1, which was chosen here as a control on sequence conservation, were individually reported to be immunoreactive and showed interesting potential in discriminating between the sera of donors who recovered from a *B*. *pseudomallei* acute infection and healthy negative controls (seronegative and seropositive individuals) in ELISA tests^8^,^9^,^10^,^11^.

### Microarray tests and diagnostic performance

A peptide microarray, composed of the epitopes reported in Table 1, was tested for its ability to diagnose *Burkholderia cepacia* complex (BCC) infection detecting peptide-specific IgG in the serum of infected CF patients. Since CF patients are often affected by multiple respiratory tract infections/colonization we also checked the cross-reactivity of the peptide microarray with a set of CF patients sera that were diagnosed as BCC negative while positive for other common CF pathogens such as *P*. *aeruginosa*, *A*. *xylosoxidans and S*. *maltophilia* (see details in the Supplementary Information. Table S1).

Epitopes reported in Table 1, terminated by a spacer and a cysteine residue, were arrayed on a silicon slide coated with a maleimido-functionalised polymer to promote the oriented immobilisation of peptides *via* specific thiol addition to maleimides on a modified copoly(DMA-NAS-MAPS)^18^ coated surface. The arrays were probed with 14 serum samples from BCC positive CF patients, diagnosed by microbiological culture and MALDI-TOF spectrometry and further characterized by sequencing for genomovar identification; 11 sera from CF individuals that were negative for BCC (although positive for other respiratory tract pathogens) and 14 healthy donors (see details on samples in the Methods section and in the Supplementary Table S1). The sera from FC patients used for this study are relative to all samples suitable for testing collected in a 1 year timespan at Fondazione IRCCS Ca’ Granda, Ospedale Maggiore Policlinico, Milan. The details for microarray production are reported in the Methods section.

**Figure 3:**
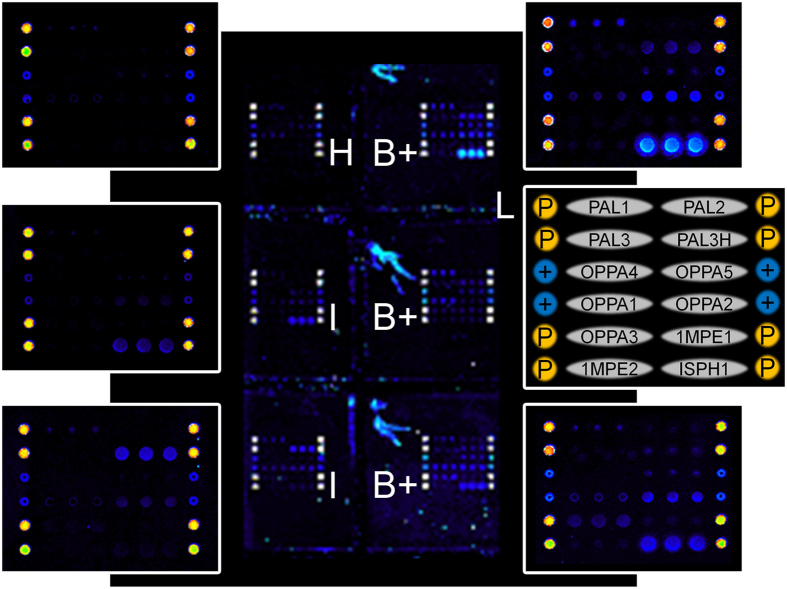
Representative fluorescence results of the peptide microarray. Each subarray is composed of the 12 peptides spotted in triplicates, 8 positional control spots (P) and 4 positive control spots (+), as it is shown in the legend (L). Each slide is spotted with 16 subarrays and incubated with 16 samples using a multiwell silicon chamber. At the centre, a portion of the slide displaying 6 adjacent subarrays that were incubated with a healthy control (H), two BCC negative controls (I) and three BCC positive patients (B+). Five representative subarrays are magnified.

The peptide-specific IgG content in each serum was evaluated by fluorescence detection using an anti-human IgG labelled with the dye Cy3. A representative microarray is reported in Fig. 3.

Healthy controls (labelled H) typically provided negligible fluorescence signals (top left array), CF patients positive for BCC (right arrays, labelled B+) showed patterns of fluorescence signals (corresponding to seroreactive peptides) that were significantly different from patterns detected in BCC negative patients (left arrays, labelled I). The ability of each epitope to distinguish between controls and patients was evaluated performing the unpaired t Test (significant if all p values <0.05) on the peptide-specific fluorescence signals detected in healthy individuals, BCC positive patients (14 samples) and the control group of CF patients affected by pathogens other than BCC (11 samples). Accuracy of discriminating between BCC infection vs healthy controls was evaluated quantitatively for each probe by a Receiver Operating Characteristic (ROC) curve analysis.

A summary of the diagnostic performance for each peptide of the array is reported in Tables 2 and S2 in the Supplementary Information, while detailed histogram representations of the t Test analysis are shown in Supplementary Figures S2 and S3.

Nine probes were found to be significantly serodiagnostic, allowing to distinguish BCC positive individuals from healthy controls (all p-values <0.05), seven of them providing 100% sensitivity and specificity (Area under the ROC curve = 1).

Extending the comparison to CF infected controls, eight probes are diagnostic for BCC and lack of cross-reactivity with the other common CF pathogens (Area under the ROC curve = 1 for 3 probes and >0.92 for 5 probes, see details in Supplementary Information, Table S2) demonstrating the high specificity of the epitopes for BCC infection detection. The peptide chemoselective immobilization strategy here adopted resulted to be critical for the analytical outcomes and the test diagnostic accuracy.

### Performance analysis

**Figure 4:**
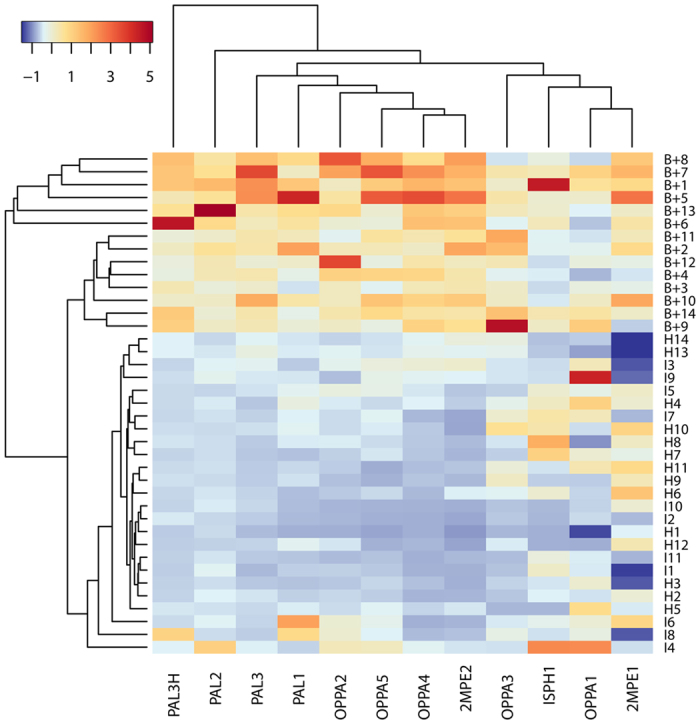
Heatmap of fluorescence intensity built after data clustering. All the data collected from the microarray platform test are reported as subdivided by probes (X axis) and donors (Y axis). Controls (H and I classes) were randomized before analysis and assigned an arbitrary donor number, while signal intensities for B+ patients are relative to specific donors. The color code follows an adimensonal scale (due to the standardization of signals). Negative values are displayed in blue gradient and positive values are displayed in yellow/red gradient. The distances and relations between clusters of probes and patients are expressed with dendrograms on their corresponding axis.

To provide a semi-quantitative evaluation of the performance of the microarray, a comprehensive cluster analysis of all data was carried out, collecting and comparing for each probe the fluorescence of *BCC* positive patients (B+) versus randomized controls. The analysis is presented in Fig. 4. Controls were randomized by assigning each spot (probe fluorescence value) to a donor at random for each control group (Healthy (H) or Infected (I)). This was made necessary by the fact that we did not know the identities of the donors due to privacy legal requirements and to the difficulties in collecting large sets of samples for CF infections. The fluorescence data were standardized for each probe, in order to minimize the statistical influence associated with extreme data points in the distribution (see Methods).

To sort the data in terms of similarity between patient profiles and probe performance, we clustered the standardized intensities grouping probes *vs*. patients using a hierarchical approach based on Manhattan distance and average linkage (see Methods). We expressed the relations between patients and probes, as well as the performance of the method with a clustered heatmap of signal intensity (Fig. 4). Concerning the probes (X-axis), it is evident that peptides ISPH1, OPPA1 and 2MPE1, and partially OPPA3 have poor or absent discrimination potential for B+ patients compared to controls as shown by the presence of positive values scattered across all serum samples. They are thus gathered in a separate cluster.

All other probes are closely related in their discrimination potential. In particular, probes OPPA4 and 2MPE2 optimally discriminate B+ cases vs. controls. PAL3H and PAL2 are considered as outgroups indicating a similar discrimination pattern and positive performance. All other probes from PAL and OPPA antigens are joined in the same cluster with similar response.

Considering the donors (Y-axis), the cluster analysis produces three main groups. The first one gathers the controls, without noticeable difference between healthy and infected donors, while the second one is composed of patients B + 8, B + 7, B + 1, B + 5, B + 13, B + 6, having a very pronounced response as a common characteristic. The third group is composed of all other B+ patients, positively discriminated by the probes against the controls.

A second test restricted to the B+ population was designed to verify whether the probes are sensitive enough to discriminate between bacterial subtypes, separating the different BCC genomovars.

**Figure 5:**
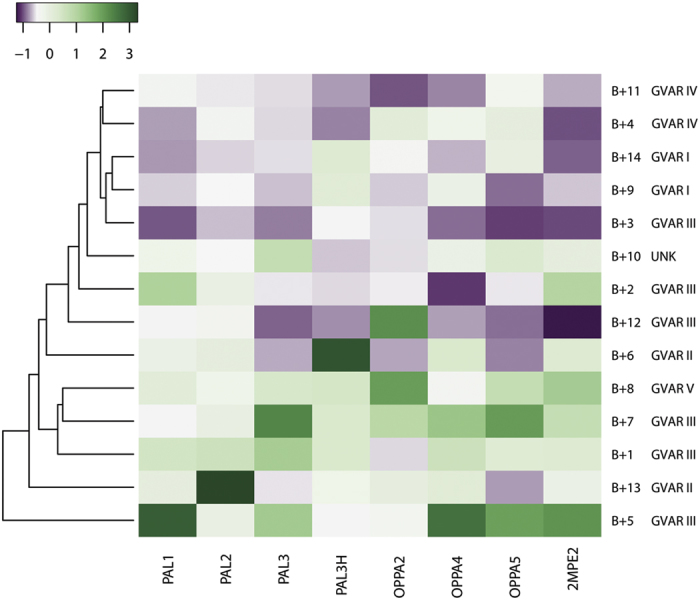
Cluster analysis of responding probes on B+ patients. In this heatmap the dataset was restricted solely to the B+ patients (Y axis) and the probes which demonstrated discriminating potential for BCC infections (X axis). The data was standardized and the color code follows an adimensional scale emphasizing the differences. The distances and relations between clusters of patients are expressed with dendrograms. Each donor is labelled with the corresponding genomovar (GVAR) of BCC infection. UNK is used to label the patient infected with BCC of unknown type.

The results of this test are shown in Fig. 5. We kept the B+ patients and removed the unresponding probes as they eventually would not be part of a diagnostic platform. The discrimination of signals into genomovars is based on much more subtle patterns than the clustering of positives vs negatives. Our aim here is to test whether a trend can be defined from the microarray data. As a relevant caveat, it is important to note that a much wider dataset would be required for a full statistical analysis aimed at setting a reference for each specific genomic variant. Herein, our analysis is aimed at returning significant observable trends.

Using the same setup as for the previous analysis, the clustering process was successful in combining into separate groups the fluorescence from genomovar IV and genomovar I. There is only one patient infected with a genomovar V *BCC*, so that, expectedly, the clustering process is ineffective for this subtype. Genomovars II and III feature highest fluorescence intensity in their response and, according to the dendrogram, appear to be more related to each other then the rest. We tried different setups and strategies for the cluster analysis (Euclidean distance, different clustering linkage), to test the trend shown in Fig. 5 for robustness. Depending on the strategy, the cluster analysis is able to distinguish different genomovars with consistent results. For instance, with a single variation in the clustering procedure (agglomeration of clusters by WPGMC algorithm), it was furthermore possible to correctly group and sort patients for genomovars I, II and IV (data not shown). This preliminary analysis on a limited number of patients supports the feasibility of extending the statistical analysis to a wider population of patients, in order to test the possibility of improving the sensitivity of the test to even predict the bacterial subtype.

## Discussion

The genomic revolution has had a deep impact on life sciences in general and on immunology in particular. The availability of entire bacterial genomes and the development of high-throughput computational and experimental analysis tools have made it possible to rapidly and efficiently identify pathogenic protein Antigens (Ags) that may represent reactive candidates for the development of vaccines or diagnostic probes.

In parallel, progress in protein structural knowledge has favored the growth of structure-based Ag design, pushing the expansion of Structural Vaccinology, a new driving force that is being exploited for vaccine and immunodiagnostic development.

In this work, we have developed and tested a novel approach for the generation of new, rapid, efficient and cost-effective immunodiagnostic tools that integrate structural computational biology with advanced microarray technologies to diagnose *BCC* infections from the analysis of serum samples from Cystic Fibrosis patients. Importantly, our method and tools showed a high degree of reliability and sensitivity, being able to discriminate *Burkholderia* infections even in the presence of several related respiratory superinfections, which is indeed a common, and largely unsolved, medical case in CF patients.

We designed our platform as an intrinsically multiplex approach, where different probes (corresponding to different epitopes from different conserved *Burkholderia* antigens) are simultaneously interrogated. In this prototype, we started from 12 different epitopes (probes) selected from 4 antigens that are well conserved among *BCC* members, at the same time being poorly conserved in the other CF co-infecting microorganisms considered. The epitopes were designed starting from the knowledge of the recently solved 3D structures of antigenic proteins from *B*. *pseudomallei*, which showed seroreactivity in preliminary ELISA tests^8^,^9^,^10^ (Fig. 2).

Summarizing, when integrated into our microarray platform, seven probes from antigens BPSL2765, BPSS2141 and BPSL1050 displayed a full discrimination potential for infected sera (AUC = 1, Table 2), and an eighth one (PAL1) showed interesting potential, supporting the potential of our diagnostic system for development and testing on a larger scale using larger sets of clinical samples.

In the case of seven probes, we obtained AUC values of 1 due to the fact that none of the healthy controls matched the fluorescence signal of affected individuals, indicating a high discrimination potential. A high score outcome is to be expected in small sample sets, such as in this case (39 subjects). However, the number of probes with a good discrimination potential is encouraging for future tests and clinical applications of this platform. Each AUC value with 95% CI is reported in the Supplementary Information (Table S2). It is worth noting here that such limitations are intimately connected to the fact that CF is a rare disease, and *Burkholderia* infections represent a subgroup of the CF affected population. Indeed, extensions of the studies to include more patient samples from different centers would be needed to fully establish the clinical relevance of our approach. Such steps are currently being organized.

The positive control on epitope conservation (epitope ISPH1 from antigen BPSL0919) returned as expected a cross-reactive response with controls, and only two other epitopes scored poorly with significant cross reactivity (Table 2, Fig. 4). The redundancy given by the use of many probes guarantees a high specificity for the test, producing a specific pattern given by the combined probe intensities (Fig. 4), which represents a “fingerprint” of the response.

Moreover, our platform demonstrated high diagnostic specificity and sensitivity even in the face of bacterial co-infections, for which we observe no cross-reactivity, providing high content results using just 1 microliter of serum, in a total analysis time of 3 hours.

While other methods based on PCR and cell culture techniques are available for CF opportunistic infections, our work is unique in that it directly advances the integration of genomic analysis, atomic-resolution computational epitope design, peptide synthesis and microarray technology, into a new and efficient tool for serological antibody screening in BCC infections that is reasonably cheap and usable in any laboratory. Moreover, further clustering analysis of the results defines trends that support the possibility to characterize *BCC* infection even at the level of bacterial genotypes (genomovar).

In fact, the various fluorescence signals from different probes define a distinct “fingerprint” for each patient. With a multiplex approach based on baits designed from a common cognate pathogen (*B*. *pseudomallei*), our aim was to exploit the adaptability and specificity of the polyclonal response against their epitope^19^ to modulate a differential Ag-antibody response among the different bacterial variants. Thus, one additional use of this fingerprint is to detect and differentiate this specific response. Based on our results, we successfully sorted patients affected by genomovars I and IV with respect to the rest of the samples with a hierarchical cluster analysis based on average fingerprint similarity (Fig. 5).

We acknowledge that the sample set used in this study is inevitably too limited to allow a significant and quantitative correlation between a patient’s fingerprint and a specific genomovar prediction. The limited sampling is strictly tied to the rare prevalence of FC in European population (1 in 13000 people or less)^20^, meaning that further trials and testing of our technology will need cooperation with a panel of FC institutions. For this reason, at current state the clustering of genomovars reveals no more than an interesting trend, providing qualitative support to the feasibility of extending our serological procedure to larger clinical sera collections.

Many investigations support the use of serological diagnostics in CF to alert clinicians to seek for other evidence of infection in the event of negative cultures^21^, for improved understanding of the colonisation/infection process^22^, and indications on the necessity for antibiotic treatment to prevent chronic infection^23^. However, only a few examples of serological diagnostic approaches applied to CF patients infected by *BCC* and other associated pathogens are present in the literature, due to the paucity of available recombinant antigens and poorly standardised methods. Moreover, the inherent complexity of the multiplex CF respiratory infections require several ELISA tests to be run for each plausible pathogen, making the process to monitor antibody response for the entire spectrum of CF’s associated infections expensive and cumbersome. Conversely, the integration of additional diagnostic probes may transform this peptide array into an even more complete and versatile platform, while keeping (or even improving) advantages like high-throughput testing and cost effectiveness. One further advantage of the peptide array approach is that the detailed interrogation of immunity provided by the combined use of multiple, well characterized synthetic epitopes reduces the susceptibility of serology to cross-reactivity and false negatives offering the potential for superior test accuracy compared to full-antigen based assays which employ pathogen extracts, lipopolysaccharides or proteins used as single antibody baits.

In addition, peptides can be synthesized introducing non-natural functionalities such as linkers, or covalent bonding. Such strategies have a dual purpose: they assist the production of peptides mimicking more closely the native spatial conformation of epitopes, and they aid a chemoselective and oriented microarray immobilization on purposely designed microarray surfaces, favoring in turn a better and specific antibody recognition.

These technical hurdles and the improvements here proposed are not specific of BCC infection-screening alone. For instance, there are similar issues with the diagnosis of melioidosis and glanders, caused by *B*. *pseudomallei* and *B*. *mallei* respectively. In perspective, considering the conservation of epitope sequences across the *Burkholderia* genus, we anticipate a possible use of the peptide array presented here for the screening of multiple *Burkholderia* infections.

In the context of the diagnosis of melioidosis, which is characterized by very dangerous and acute episodes, one cannot just rely on the screening of IgG molecules. In our case, we focused the present investigation on the detection of IgGs only since the sera we analyzed were from patients known to be affected by chronic lung infection. In addition, the relevance of IgA vs IgG serum levels in CF patients affected by BCC, in both intermittently and chronically colonised patients, is still unclear^24^. However, we expect the peptide array platform to be perfectly suited to monitor IgM and IgA response as well, to monitor the first phases of an infection and to establish its onset.

For the aforementioned reasons, we consider our method ready to be carefully tested on a larger clinical scale. In parallel, we expect the method validated here for the specific case of *BCC* in CF infections to be considered as general, with the potential to be extended to other diseases and meet other diagnostic needs.

## Materials and Methods

### Reagents

Reagents for peptide synthesis were from Iris Biotech (Marktredwitz, Germany). Other chemicals were from Sigma-Aldrich (St. Louis, MO, USA) if not stated otherwise. Goat anti-human IgG labeled by Cy3 were obtained from Jackson Immunoresearch (West Grove, PA, USA). Silicon slides were from SVM (Sunnyvale, CA).

### Epitope prediction and design

We first verified that selected antigens BPSS2141, BPSL2765 and BPSL1050 are well conserved among *BCC* members, and at the same time they are poorly conserved in the other microorganisms considered, which represent common co-infecting pathogens in CF patients (see Supplementary Figure S1). For this task, we used the web-based, freely available resources at EnsemblGenomes^17^, searching and aligning our query sequences (antigens and epitopes) using BLASTp.

The epitopes were selected and designed using the MLCE prediction method (Matrix of Local Coupling Energies, MLCE^25^,^26^, available also as a public web-based service http://bioinf.uab.es/BEPPE)^8^,^9^,^11^,^27^,^28^. MLCE is based on the idea that structural dynamics determines why specific parts of a protein antigen may bind to antibodies and/or tolerate mutations that allow the pathogen to escape protective immune responses, all typical traits of B-cell epitopes. These salient aspects are captured in MLCE by the concept that epitope regions correspond to non-optimized intramolecular interaction-networks in the Ag structure.

The structure of antigen BPSL0919 was reconstructed by homology with protein IspH1 from *E*. *coli*. (PDB ID: 3F7T, 58% S-W identity and 83% S-W similarity^29^ with BPSL0919 protein sequence. The initial model was built using Modeller 9^30^, and further refined via MD simulation. The simulation and analysis were performed using the GROMACS 4.54 software package^31^, GROMOS 53A6 force field^32^ and the SPC water model^33^. The antigen was simulated in NPT conditions for 50 ns at 300 K. Epitope predictions were carried out using the MLCE prediction method^25^,^26^ on the representative structure of the most populated structural cluster obtained using the method developed by Daura *et al*.^34^.

### Peptide synthesis

All linear peptides were synthesized by stepwise microwave-assisted Fmoc-SPPS on a Biotage ALSTRA Initiator+ peptide synthesizer according to well-established protocols^8^,^9^,^10^,^11^,^35^. Briefly, peptides were assembled on a 2-CTC resin. Chain elongation was performed by iterative cycles of amino acids coupling (using Oxyma/DIC as activators) and Fmoc-deprotection using a 20% piperidine solution in DMF. Upon complete chain aseembly, peptides were cleaved from the resin using a 2.5% TIS, 2.5% thioanisole, 2.5% water, 92.5% TFA mixture. Crude peptides were then purified by preparative RP-HPLC. MS analysis was performed separately on purified material.

In the case of conformational epitopes (PAL2, OPPA1), we used additional suitable spacers (either PEG or additional Gly residues) to combine different parts of the epitope sequences such that the relative distance between the terminals could reproduce the average one observed in the cognate antigen (Peri *et al*.^28^).

All epitopes were conjugated to PEG units as spacers to separate the probes from the microarray surface.

### Peptide microarrays

Silicon slides were coated by copoly(DMA-NAS-MAPS) according to the protocol described in^36^. Briefly, silicon slides were immersed in a polymer solution (1% w/v in 0.9 M (NH_4_)_2_SO_4_) for 30 min, then rinsed with water, dried under nitrogen and cured for 15 min under vacuum at 80 °C. To functionalize slides with maleimido groups, slides were immersed in a solution containing 6 mM of N-(2-Aminoethyl)maleimide trifluoroacetate salt and 7 mM N,N-Diisopropylethylamine in dimethyl formamide (DMF) for 5 hours at 40 °C. Slides were then throughly washed in DMF and water and dried under a stream of nitrogen.

Peptides were first dissolved with 50% acetonitrile solution (v/v, in Milli-Q water) to 10 mg/mL stock solution and then diluted to 2 mg/mL into the printing buffer (25 mM Na/Acetate pH 4.8, 15 mM trehalose). Microarrays were prepared using a non-contact sciFLEXARRAYER S3 (Scienion Co., Berlin, Germany) spotter. All the peptide samples were printed in triplicates, each sub-array has positional controls (Cy3-streptavidin) and positive controls (human IgG at the concentration of 0.2 mg/mL). Sixteen arrays were spotted on each silicon slide corresponding to the sixteen compartments created by NEXTERION^®^ IC-16 sixteen well Incubation Chamber from Schott (Jena, Germany). Printed slides were placed in a humid chamber and incubated overnight at room temperature. Then they were blocked with 2% w/v BSA in PBS for 1 h, washed with water and dried under a stream of nitrogen.

Serum was diluted 1:100 in LowCross-Buffer^®^ from Candor (Wangen, Germany) and 40 μL were added into each microarray well, incubated for 60 min on a shaker (150 rpm, 22 °C). BCC positive, BCC negative and healthy sera were incubated in parallel up to 16 samples per slide. Negative controls included blank arrays incubated only with the secondary antibody and with incubation buffer.

The microarray slide was then rinsed for 3 times with washing buffer (0.05 M Tris/HCl pH 9, 0.25 M NaCl, 0.05% v/v Tween 20) and PBS and incubated with 40 μL of 1 ug/mL Cy-3 labelled goat anti-hIgG for another 60 min followed by the same washing steps as described above.

Fluorescence was detected by a ProScanArray scanner (PerkinElmer, Boston, MA) using 70% photomultiplier (PMT) gain and laser power.

Fluorescence intensities were analyzed using the QuantArray software from PerkinElmer. The fluorescence intensities were corrected for spot-specific background, values for replicate spots were averaged. t Student tests over the groups of samples and ROC curve analysis were performed using Prism 6 from GraphPad.

### Cluster analysis

The standardization of data was carried out on the raw fluorescence intensities collected from the microarray tests using median and MAD (Median Absolute Deviation) of the distribution *ƒ*(*Z*) from each probe.

The standardized value for each data point associated to the same probe is calculated as:


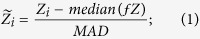


The cluster analysis was carried out on the standardized dataset using a bottom-up hierarchical approach based on Manhattan^37^ metric to define the distance *d*(*x*_*i*_, *y*_*j*_) between points *x*_*i*_ ∈ *X and y*_*j*_ ∈ *Y*, and average linkage to define the distance *D*(*X*, *Y*) between clusters:


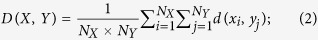


Statistical analysis were performed using R^38^.

### Samples

Serum samples drawn from healthy controls and CF patients were from the biobank of Fondazione IRCCS Ospedale Maggiore Policlinico, Milano. All samples were collected, analyzed and stored according to the rules of the local ethical comittee. Table S2 in the Supplentary informations summarizes the details on the serum samples used in this work. The microbiological evaluation was performed on sputum samples from patients followed at CF centre of Milan, routinely processed and investigated, according to CF microbiological standard procedure, every 3 months over a one year period. Samples, obtained from the low respiratory tract, were firstly mixed with dithiothreitol at a 1:1 dilution to reduce viscosity, and then incubated at 37 °C for 30 minutes. Treated samples were plated on several media and plates were incubated at 37 °C for 72 hours. In particular, *B*. *cepacia* complex strains grow on *Burkholderia Cepacia* Selective Agar (BCSA) and on MacConkey agar. All presumptive *B*. *cepacia* complex colonies were firstly identified by Maldi-Tof spectrometry and successively spread over blood agar for 24 h and stored at −80 °C in order to permit further examinations to have genomovar identification. The *rec*A gene of the *B*. *cepacia complex* strains was examined, PCR primers used were: BCR1:TGACCGCCGAGAAGAGCAA and BCR2:CTCTTCTTCGTCCATCGCCTC.

Analysis of the *rec*A gene of the *B*. *cepacia* complex provides a rapid and robust nucleotide sequence-based approach to identify and classify this taxonomically complex group of opportunistic pathogens^39^. The sequences were submitted to GenBank as an aligned set using BLAST, in order to get the identification.

A document informing that samples could be used for research purposes has been signed by all subjects whose serum was used in the exepriments. Experiments have been carried out following current guidelines and regulations.

## Additional Information

**How to cite this article**: Peri, C. *et al*. Evolving serodiagnostics by rationally designed peptide arrays: the *Burkholderia* paradigm in Cystic Fibrosis. *Sci. Rep*. **6**, 32873; doi: 10.1038/srep32873 (2016).

## Supplementary Material

Supplementary Information

## Figures and Tables

**Figure 1:**
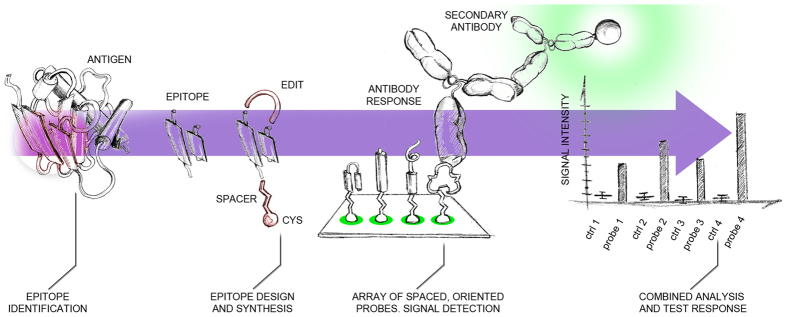
Pictorial scheme of the method’s workflow. Starting from an antigen’s 3D structure, epitope identification is carried out by MLCE computational predictions. The epitopes are modified at the structural level in order to be synthesized as peptides and spotted as spaced and oriented baits. The platform is interrogated with patients’ antisera and detection occurs by means of secondary antibodies. The signal intensity is subsequently acquired and analyzed for a test response.

**Table 1:** Epitopes and sequences.

ANTIGEN/PDB CODE	EPITOPE NAME	PROBE SEQUENCE
BPSL2765/4B5C	PAL1	Cys-PEG-(144)GKEKPVALGHDEASWAQNRRADLVYQ
BPSL2765/4B5C	PAL2	Cys-PEG-(132)GVGDAQMEAV-PEG-(89)YLKSHPQRHIL
BPSL2765/4B5C	PAL3	Cys-PEG-PEG-(72)SYSVQDQYQALLQQHAQYLK
BPSL2765/4B5C	PAL3H	Cys-PEG-PEG-(72)SYSVQDQYQALLQQHAQYLK-[Lys-N3]QAE[Prg]
BPSS2141/3ZS6	OPPA1	Cys-PEG-PEG-(106)KAPDT*gg*(184)KTEVPVSY
BPSS2141/3ZS6	OPPA2	Cys-PEG-PEG-(357)GVKGVQRPFTPDWA
BPSS2141/3ZS6	OPPA3	Cys-PEG-PEG-(488)AEANQKLDDGARAALLTQAHDLA
BPSS2141/3ZS6	OPPA4	Cys-PEG-PEG-(121)WSNGQPVTAADFVYAWQR
BPSS2141/3ZS6	OPPA5	Cys-PEG-PEG-(299)ELRPGLQLATYYYYLK
BPSL1050/2MPE	2MPE1	Cys-(O2OC)2-(38)VGYGGHGHPTQVRIVAPHAEHVRGYAH
BPSL1050/2MPE	2MPE2	(84)DGAARFERYLAALPRKLAAWENARGVDFGSRTQADAL-(O2OC)2-Cys
BPSL0919/3F7T*	ISPH1	Cys-(O2OC)2- (304)ALEGIEENVSFPLPRGL

At the heart of each probe is a synthetic peptide corresponding to an immunoreactive epitope from *B*. *pseudomallei* antigens. For each epitope is shown its corresponding antigen in form of its UniProt ID^40^ and RCSB PDB^41^ code. The last column includes the amino acid sequence and the chemical modifications carried out for synthesis. All sequences are based on linear epitopes except for epitope PAL2 and OPPA1, which are based on conformational antibody binding sites. *BPSL0919 does not feature a structural record in the PDB, thus the antigen was modelled based on homology with PDB ID 3F7T, corresponding to *E*. *coli* IspH.

**Table 2:** Evaluation of the diagnostic performance of the peptide array.

Probe	AUC: Area Under the ROC Curve (B+): BCC positive patients (H): healthy controls	Unpaired t Test ns: not significative Significative: p < 0.05; * = p < 0.05; ** = p < 0.01; *** = p < 0.001; **** = p < 0.0001. (B+): BCC positive patients (H): healthy controls (I): BCC negative	
	AUC B+ vs H	B+ vs H	B+ vs I
**PAL1**	0.9505	**	ns
**PAL2**	1	**	*
**PAL3**	1	****	***
**PAL3H**	1	***	**
**OPPA1**	0.6786	ns	ns
**OPPA2**	1	***	**
**OPPA3**	0.8352	*	**
**OPPA4**	1	****	****
**OPPA5**	1	****	**
**2MPE1**	0.6071	ns	**
**2MPE2**	1	****	****
**ISPH1**	0.6684	ns	ns

Each probe is followed by a column describing the AUC measure of performance for the classification of donors as BCC positive or negative subjects. 7 probes out of 12 reach a full discrimination potential (AUC = 1). The Unpaired t Test was used as a measure of statistical significance. The test was carried out on the performance of discrimination against a pool of healthy controls (B+ vs H) and a pool of infected FC patients negative to BCC screening (B+ vs I).
