# Supplementary material for: Evolving serodiagnostics by rationally designed peptide arrays: the Burkholderia paradigm in Cystic Fibrosis
Source: Sci Rep. 2016 Sep 12;6:32873. doi: 10.1038/srep32873 (PMC5018727; doi:10.1038/srep32873)
Supplement: Supplementary Information [file srep32873-s1.pdf]

# Evolving serodiagnostics by rationally designed peptide arrays: the *Burkholderia* paradigm in Cystic Fibrosis

Claudio Peri[1], Alessandro Gori[1], Paola Gagni[1], Laura Sola[1], Daniela Girelli[2], Samantha Sottotetti[2], Lisa Cariani[2], Marcella Chiari[1], Marina Cretich[1*], and Giorgio Colombo[1]*

[1]Istituto di Chimica del Riconoscimento Molecolare, ICRM, CNR. Via Mario Bianco 9, 20131, Milano (Italy)

[2]Cystic Fibrosis Microbiology Laboratory, Fondazione IRCCS Cà Granda, Ospedale Maggiore Policlinico, Milano (Italy)

## SUPPLEMENTARY INFORMATION

| ANTIGEN | ORGANISM | #num | E-value | al. Length | IDENTITY (%) | GENOMIC LOCATION | GKEKPVALGHDEASWAQNRRADLVYQ | GVGDAQMEAV-YLKSHPQRHIL | SYSVQDQYQALLQQHAQYLK |
|---|---|---|---|---|---|---|---|---|---|
| BPSL2765 | B. thailandensis | 1 | 2.40E-97 | 170 | 98.2 | 1:217180 to 217689 (+) | GKEKPVALGHDEASWAQNRRADLVYQ | GVGDSQMEAV-YLKSHPQRHIL | SYSVQDQYQPLLQQHAQYLK |
| (170 res) | B. mallei | 1 | 4.30E-98 | 169 | 100 | 1:873919 to 874425 (-) | GKEKPVALGHDEASWAQNRRADLVYQ | GVGDAQMEAV-YLKSHPQRHIL | SYSVQDQYQALLQQHAQYLK |
| | B. cepacia | 1 | 1.80E-83 | 169 | 84 | 1:1127705 to 1128211 (+) | GKEKPQATGHDEASWAQNRRADLVYQ | GVNDSQMEAV-YLKSHPQRHVL | SYSVKDEYQPLMQQHAQYLK |
| | | 2 | 7.80E-55 | 130 | 73.8 | 2:29687 to 30076 (-) | GKEKPVALGHDEEAWAQNRRADLVYR | GVPATQLEAV-YLRSHPARHVL | QYSVKPEFQSLLQAHADYLR |
| | B. multivorans | 1 | 2.70E-68 | 135 | 88.9 | BMD22_012: 63223 to 63627 (-) | GKEKPQATGHDEASWAQNRRADLVYQ | GVADSQMEAV-YLKSHPQRHVL | SYSVKDEYQPLLQQHAQYLK |
| | | 2 | 8.50E-55 | 137 | 70.1 | BMD22_004: 109126 to 109536 (-) | GKEKPVALGHDEAAWAQNRRADLVYR | GVSASQLEAV-YLRSHPSRHVL | SYTVKPEDQSLLQAHANYLR |
| | B. cenocepacia | 1 | 5.10E-68 | 135 | 88.1 | 1: 1592797 to 1593201 (-) | GKEKPQATGHDEASWAQNRRADLVYQ | GVNDSQMEAV-YLKSHPQRHVL | SYSVKDEYQPLLQQHAQYLK |
| | | 2 | 8.70E-55 | 157 | 64.3 | 2: 1700236 to 1700706 (+) | GKEKPVALGHDEESWSQNRRADLVYR | GVQASQLEAV-YLRSHPSRHVL | QYSVKPEYQSLLQAHADYLR |
| | B. vietnamiensis | 1 | 3.80E-84 | 170 | 84.7 | 1: 809317 to 809826 (-) | GKEKPQATGHDEASWAQNRRADLVYQ | GVNDSQMEAV-YLKSHPQRHVL | SYSVKDEYQPLLQQHAQYLK |
| | B. dolosa | 1 | 3.50E-69 | 135 | 89.6 | CH482380: 2938831 to 2939235 (+) | GKEKPLATGHDEASWAQNRRADLVYQ | GVADSQMEAV-YLKSHPQRHVL | SYSVKDEYQPLLQQHAQYLK |
| | B. ambifaria | 1 | 3.10E-68 | 135 | 88.1 | 1: 927420 to 927824 (+) | GKEKPQASGHDEASWAQNRRADLVYQ | GVNDSQMEAV-YLKSHPQRHVL | SYSVKDEYQPLMQQHAQYLK |
| | | 2 | 2.30E-53 | 129 | 74.4 | 2: 719403 to 719789 (+) | GKEKPVALGHDEDSWAQNRRADLVYR | GVQATQLEAV-YLRNHPSRRVL | QYSVKPEFQSLLQSHADYLR |
| | P. aeruginosa | 1 | 8.70E-27 | 71 | 63.4 | contig00001: 7116 to 7328 (-) | GKERPVATGHDEQSWAQNRRVEL--- | GVSPAQLELV--------QRVV | n.a. |
| | A. xylosoxidans | 1 | 1.70E-29 | 79 | 64.6 | 1quiver: 5835632 to 5835868 (-) | GKEKPKATGTSEADFAENRRADIVYR | GVSDNQIETI----SHQQQTIK | n.a. |
| | S. maltophilia | 1 | 6.80E-22 | 113 | 43.4 | CP008838:27470 to 27808 (+) | GEERPVCTESNESCWSQNRRVEIVY- | GGSASQLTVV-YLRDRPSSRIT | KEDVKPEFQAIMACHAKYLR |
| | S. aureus | 1 | 1.10E-04 | 25 | 48 | CP008838:27734 to 27808 (+) | n.a. | n.a. | n.a. |
| | C. albicans | 1 | 8.40E-02 | 51 | 27.5 | cont1.27:269714 to 269866 (+) | n.a. | n.a. | n.a. |

| ANTIGEN | ORGANISM | #num | E-value | al. Length | IDENTITY (%) | GENOMIC LOCATION | KAPDT-KTEVPVSY | GVKGVQRPFTPDWA | AEANQKLDDGARAALLTQAHDLA | WSNGQPVTAADFVYAWQR | ELRPGLQLATYYYYLK |
|---|---|---|---|---|---|---|---|---|---|---|---|
| BPSS2141 | B. thailandensis | 1 | 0.00E+00 | 554 | 95.7 | 2: 2409607 to 2411268 (-) | KTPDT-TTEVPVSY | GVKGVQRPFTPDWA | AEANQKLDDGARSALLTQAHDLA | WSNGQPVTAADFVYSWQR | ELRPGLQLATYYYYLK |
| (554 res) | B. mallei | 1 | 0.00E+00 | 539 | 99.8 | DM79.Contig107: 3672229 to 3673845 (+) | KAPDT-KTEVPVSY | GVKGVQRPFTPDWA | AEANQKLDDGARAALLTQAHDLA | WSNGQPVTAADFVYAWQR | ELRPGLQLATYYYYLK |
| | B. cepacia | 1 | 0.00E+00 | 328 | 81.7 | 2: 1902510 to 1903493 (+)** | KTPQT-TTDVPVAF | GTKGVQQPFTPEWA | DEGNQKLDDQARTTLLTQAHDMA | WSNGQPVTAADFVYSWQR | ELRPGLQLATYYYYLN |
| | | 1 | 5.20E-113 | 192 | 83.9 | 2: 1903491 to 1904066 (+)** | | | | | |
| | B. multivorans | 1 | 0.00E+00 | 328 | 82.6 | BMD22_009: 193855 to 194838 (-)** | KTPQT-TTDVPVAF | GTKGVQQPFTPEWA | DEGNQKLDDKARAALLTQAHDTA | WSNGQPVTAADFVYSWQR | ELRPGLQLATYYYYLN |
| | | 1 | 2.70E-116 | 192 | 87 | BMD22_009: 193282 to 193857 (-)** | | | | | |
| | B. cenocepacia | 1 | 0.00E+00 | 328 | 81.4 | 2: 360688 to 361671 (-)** | KTPQT-TTDVPVAF | GTKGVQQPFTPEWA | EEGNQKLDDKARTALLTQAHDMA | WSNGQPVTAADFVYSWQR | ELRPGLQLATYYYYLN |
| | | 1 | 1.40E-114 | 192 | 85.4 | 2: 360115 to 360690 (-)** | | | | | |
| | B. vietnamiensis | 1 | 0.00E+00 | 328 | 80.2 | 2: 457096 to 458079 (-)** | KTPQT-TTDVPVAF | GTKGVRRPYTPDWA | DEGNQKLDDAARTALLTQAHDLA | WSNGQPVTAADFVYSWQR | ELRPGLQLATYYYYLN |
| | | 1 | 3.20E-115 | 192 | 85.9 | 2: 456523 to 457098 (-)** | | | | | |
| | B. dolosa | 1 | 0.00E+00 | 328 | 82.6 | CH482381: 1700792 to 1701775 (-)** | KTPQT-TTDVPVAF | GTKGVQQPFTPEWA | DDGNQKLDDSARAALLTQAHDAA | WSNGQPVTAADFVYSWQR | ELRPGLQLATYYYYLN |
| | | 1 | 8.40E-116 | 192 | 86.5 | CH482381: 1700219 to 1700794 (-)** | | | | | |
| | B. ambifaria | 1 | 0.00E+00 | 328 | 81.4 | 2: 1854672 to 1855655 (-)** | KTPQT-TTDVPVAF | GTKGAQQPFTPDWA | DEGNQKLDDKARTALLTQAHDTA | WSNGQPVTAADFVYSWQR | ELRPGLQLATYYYYLN |
| | | 1 | 5.20E-114 | 192 | 85.4 | 2: 1854099 to 1854674 (-)** | | | | | |
| | P. aeruginosa | 1 | 1.90E-03 | 60 | 28.3 | contig_61: 6920 to 7099 (-) | n.a. | n.a. | n.a. | n.a. | n.a. |
| | A. xylosoxidans | 1 | 2.20E-12 | 67 | 35.8 | 1quiver: 4914519 to 4914719 (+) | n.a. | n.a. | n.a. | FHDGSPFTADDVIFSWKR | n.a. |
| | | 2 | 1.00E-10 | 82 | 34.1 | 1quiver: 4660221 to 4660466 (+) | n.a. | n.a. | n.a. | FSNGQPFTAQDVLFTFCR | n.a. |
| | S. maltophilia | 1 | 2.00E-02 | 41 | 29.3 | contigID18:206809 to 206931 (+) | n.a. | n.a. | n.a. | n.a. | n.a. |
| | S. aureus | 1 | 7.50E-29 | 100 | 48 | CPJO01000001:88198 to 88497 (+)** | n.a. | n.a. | KVARTKL---------------- | WSNGDKVTAQDFVYAWRK | n.a. |
| | | 1 | 1.60E-08 | 71 | 32.4 | CPJO01000001:89134 to 89346(+)** | | | | | |
| | | 1 | 7.70E-08 | 63 | 28.6 | CPJO01000001:88510 to 88698 (+)** | | | | | |
| | C. albicans | n.a. | n.a. | n.a. | n.a. | n.a. | n.a. | n.a. | n.a. | n.a. | n.a. |

| ANTIGEN | ORGANISM | #num | E-value | al. Length | IDENTITY (%) | GENOMIC LOCATION | VGYGGHGHPTQVRIVAPHAEHVRGYAH | **DGAARFERYLAALPRKLAAWENARGVDFGSRTQADAL** |
|---|---|---|---|---|---|---|---|---|
| BPSL1050 | B. thailandensis | 1 | 9.30E-78 | 126 | 90.5 | 1: 2036742 to 2037119 (-) | VGYGGHGHPTQVRIAAPPAEHVRGYAH | DGAARFERYLAALPRKLPAWENARGIDFGSRTQTDAL |
| (126 res) | B. mallei | 1 | 2.30E-85 | 126 | 99.2 | DM79.Contig107: 1145430 to 1145807 (+) | VGYGGHGHPTQVRIVAPHAEHVRGYAH | DGAARFERYLAALPRKLAAWENARGVDFGSRTQADAL |
| | B. cepacia | 1 | 8.10E-57 | 125 | 69.6 | 1: 2900837 to 2901211 (-) | VPYGGHGHPTRVQIRSAPHEHVSGFVH | DGPQRFEHYLAALPRKLGAWQGARDIDLASRTQADPL |
| | B. multivorans | 1 | 3.70E-59 | 125 | 72.8 | BMD22_006: 166364 to 166738 (+) | VPYGGHGHPTRVRIRSAPHEHVSGFAH | DGAQRFEHYLAALPKKLNAWQGARDIDLASRTQADPL |
| | B. cenocepacia | 1 | 1.40E-58 | 125 | 72 | 1: 28637 to 29011 (+) | VPYGGHGHPTCVQIRSAPHEHVSGFAH | DGPARFEHYLSALPRKLDAWQGARDIDLASRTQADPL |
| | B. vietnamiensis | 1 | 5.70E-56 | 125 | 68.8 | 1: 2746743 to 2747117 (+) | VPYGGHGQPTRVHISSAPHEHVSGFAY | DGAARFEHYLSALPKKLNAWESARDIDLASRTQEEPL |
| | B. dolosa | 1 | 1.60E-58 | 125 | 71.2 | CH482380: 1144870 to 1145244 (-) | VPYGGHGHPTCVRVRSAPHEHVSGFAH | DGRARFDRYLAALPRKLAAWQDARDIDLPSRTQAEPI |
| | B. ambifaria | 1 | 9.80E-56 | 125 | 68.8 | 1: 2557668 to 2557942 (-) | APYGGHGQPTRVRIRSAPHEHVSGFVH | DGPQRFEHYLAALPKKLGAWQGARDIDLASRTQADPL |
| | P. aeruginosa | 1 | 4.40E-02 | 28 | 42.9 | contig_1: 208802 to 208885 (-) | ----GYFRTFEQKIATPHGEEVRGYHK | n.a. |
| | A. xylosoxidans | n.a. | n.a. | n.a. | n.a. | n.a. | n.a. | n.a. |
| | S. maltophilia | n.a. | n.a. | n.a. | n.a. | n.a. | n.a. | n.a. |
| | S. aureus | n.a. | n.a. | n.a. | n.a. | n.a. | n.a. | n.a. |
| | C. albicans | n.a. | n.a. | n.a. | n.a. | n.a. | n.a. | n.a. |

| ANTIGEN | ORGANISM | #num | E-value | al. Length | IDENTITY (%) | GENOMIC LOCATION | **ALEGIEENVSFPLPRGL** |
|---|---|---|---|---|---|---|---|
| BPSL0919 | B. thailandensis | 1 | 0.00E+00 | 326 | 99.4 | 1: 1885972 to 1886949 (+) | ALEGIEENVSFPLPRGL |
| (326 res) | | 2 | 3.70E-109 | 308 | 56.2 | 2: 2441169 to 2442092 (+) | TMSGREEKVEFKLPAKL |
| | B. mallei | 1 | 0.00E+00 | 326 | 100 | DM79.Contig107: 5099949 to 5100926 (-) | ALEGIEENVSFPLPRGL |
| | | 2 | 3.20E-109 | 308 | 55.8 | DM79.Contig107: 3161748 to 3162671 (+) | TMSGREEKVEFKLPAKL |
| | B. cepacia | 1 | 0.00E+00 | 326 | 94.8 | 1: 2777678 to 2778655 (+) | ALDGIEENVSFPLPRGL |
| | | 2 | 8.70E-109 | 308 | 54.9 | 2: 1891254 to 1892177 (-) | TMAGREEKVEFKLPAKL |
| | B. multivorans | 1 | 0.00E+00 | 325 | 95.1 | BMD22_006: 277818 to 278792 (-) | ALEGIEENVSFPLPRGL |
| | | 2 | 9.40E-109 | 303 | 56.1 | BMD22_009: 205118 to 206026 (+) | TMAGREEKVEFKLPAKL |
| | B. cenocepacia | 1 | 0.00E+00 | 326 | 95.4 | 1: 142815 to 143792 (-) | ALEGIEENVSFPLPRGL |
| | | 2 | 5.10E-109 | 308 | 55.5 | 2: 372150 to 373073 (+) | TMAGREEKVEFKLPAKL |
| | B. vietnamiensis | 1 | 0.00E+00 | 326 | 94.5 | 1: 2850261 to 2851238 (-) | ALEGIEENVAFPLPRGL |
| | | 2 | 6.40E-109 | 303 | 56.1 | 2: 468398 to 469306 (+) | TMAGREEKVEFKLPAKL |
| | B. dolosa | 1 | 0.00E+00 | 325 | 95.4 | CH482380: 1025820 to 1026794 (+) | ALEGIEENVSFPLPRGL |
| | | 2 | 4.40E-90 | 276 | 52.9 | CH482381: 1712463 to 1713290 (+) | TMAGREEKVEFKLPAKL |
| | B. ambifaria | 1 | 0.00E+00 | 325 | 94.5 | 1: 2437219 to 2438193 (+) | ALDGIEENVSFPLPRGL |
| | | 2 | 4.60E-110 | 308 | 55.8 | 2: 1866068 to 1866991 (+) | TMAGREEKVEFKLPAKL |
| | P. aeruginosa | 1 | 4.10E-64 | 172 | 58.1 | contig_5: 161994 to 162509 (+)** | ELEGREENITFSMPKEL |
| | | 2 | 6.10E-61 | 134 | 67.9 | contig_5: 161574 to 161975 (+)** | |
| | A. xylosoxidans | 1 | 4.50E-159 | 319 | 70.8 | 1quiver: 274951 to 275907 (+) | TMPGLEENVAFPLPKGL |
| | S. maltophilia | n.a. | n.a. | n.a. | n.a. | n.a. | n.a. |
| | S. aureus | n.a. | n.a. | n.a. | n.a. | n.a. | n.a. |
| | C. albicans | n.a. | n.a. | n.a. | n.a. | n.a. | n.a. |

Figure S1. **Epitope conservation among Burkholderia species and common FC superinfection agents**: the table is subdivided in four sections, one for each antigen. The conservation has been evaluated via BLASTp against the proteome of 11 bacteria and 1 fungus using the EnsemblGenomes database (www.ensemblgenomes.org). For each query, only clear homologues have been reported in case of strong similarities, and only the best hit in case of poor similarities. A color code is adopted to underline the similarity level: exact or almost exact matches are shown in green background. Blue background is for homologous proteins, yellow stands for poor similarity and red is for no similarity. For each genome, the table shows the number of similar proteins considered, the length of the aligned region, the percentage of identity between the aligned sequences, the genomic location on the reference genome and its orientation on the DNA strand (forward = +, reverse = -) and the epitope sequences (mutations highlighted in red). **One locus has been split by EnsemblGenomes during alignment. Multiple results here are actually a single one.

| | Infections | N | N | | |
|---|---|---|---|---|---|
| BCC positive CF patients | Genomovar I | 2 | 14 | (B+) | |
| | Genomovar II | 2 | | | |
| | Genomovar III | 6 | | | |
| | Genomovar IV | 2 | | | |
| | Genomovar V | 1 | | | |
| | UNK | 1 | | | |
| BCC negative CF patients | *P. aeruginosa* | 6 | 11 | (I) | (B-) |
| | *A. xylosoxidans* | 4 | | | |
| | *S. maltophilia* | 1 | | | |
| Healthy controls | ---- | | 14 | (H) | |

Table S1. **Details on the tested serum samples**. The screening method was tested against sera collected from 39 donors, subdivided in three main categories: 14 cystic fibrosis patients (CF)

positive to *Burkholderia cepacia* complex (BCC) infections (B+); 25 individuals negative to BCC (B-), subdivided in turn by 11 cystic fibrosis patients infected by other pathogens (I) and 14 healthy controls (H). Columns "Infections" and "N" subdivide the CF patients based on the specific BCC genomovar, if known, or other pathogenic microbes. Genomovar I-V correspond in turn to species *B. cepacia, B. multivorans, B. cenocepacia, B. stabilis , B. vietnamiensis*. UNK indicates the BCC genotype for the *Burkholderia* infected patient is unknown.

| **Probe** | **AUC: Area Under the ROC Curve and 95% Confidence Interval (95% CI)**<br>(B+): BCC positive patients, 14 samples<br>(H): healthy controls, 14 samples<br>(I): Infected individuals (Negative for Burkholderia), 11 samples | |
|---|---|---|
| | **AUC B+ vs H** | **AUC B+ vs I** |
| **PAL1** | 0.9505<br>0.8682 to 1.000 | 0.8377<br>0.6513 to 1.000 |
| **PAL2** | 1.000<br>1.000 to 1.000 | 0.9221<br>0.7746 to 1.000 |
| **PAL3** | 1.000<br>1.000 to 1.000 | 1.000<br>0.8456 to 1.000 |
| **PAL3H** | 1.000<br>1.000 to 1.000 | 0.9481<br>1.000 to 1.000 |
| **OPPA1** | 0.6786<br>0.4739 to 0.8833 | 0.5179<br>0.2874 to 0.7483 |
| **OPPA2** | 1.000<br>1.000 to 1.000 | 0.9221<br>0.8180 to 1.000 |
| **OPPA3** | 0.8352<br>0.6816 to 0.9888 | 0.9416<br>0.8553 to 1.000 |
| **OPPA4** | 1.000<br>1.000 to 1.000 | 1.000<br>1.000 to 1.000 |
| **OPPA5** | 1.000<br>1.000 to 1.000 | 0.9286<br>0.8314 to 1.000 |
| **2MPE1** | 0.6071<br>0.3872 to 0.8270 | 0.8766<br>0.7310 to 1.000 |
| **2MPE2** | 1.000<br>1.000 to 1.000 | 1.000<br>1.000 to 1.000 |
| **ISPH1** | 0.6684<br>0.4534 to 0.8833 | 0.5952<br>0.3691 to 0.8213 |

Table S2: AUC and 95% Confidence Interval ROC Curve

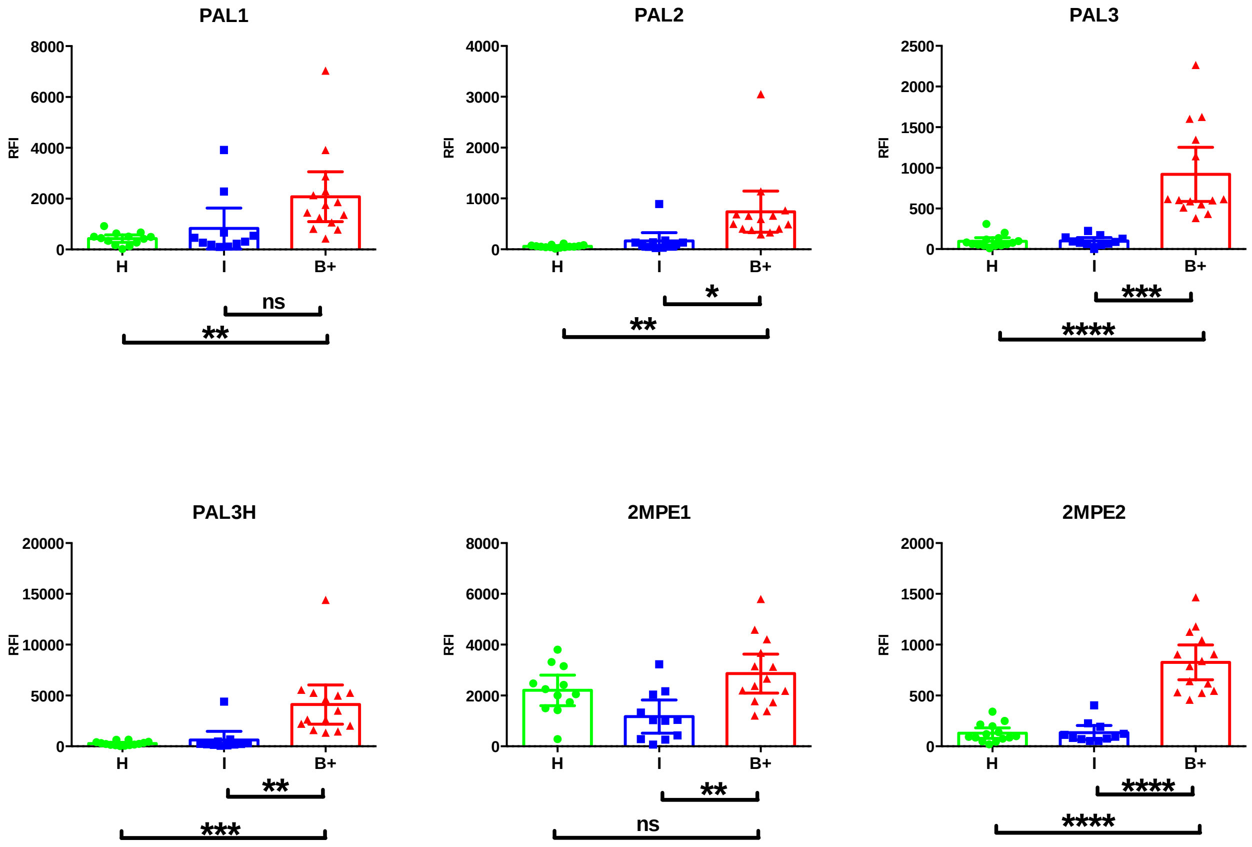


Figure S2: **Antibody recognition signals subdivided by probe and patient category (part I)**. IgG levels specific for peptides PAL1, PAL2, PAL3, PAL3H, 2MPE1 and 2MPE2 in healthy controls (H), CF patients negative for BCC and positive for other respiratory infections (I) and BCC positive patients (B+). The mean relative fluorescence intensity (RFI), detected at 70% laser power and photomultiplier and P-values of test significance are reported for each peptide.

ns: not significative . Significative: p<0.05; * = p<0.05; ** = p<0.01; *** = p<0.001; **** = p<0.0001.

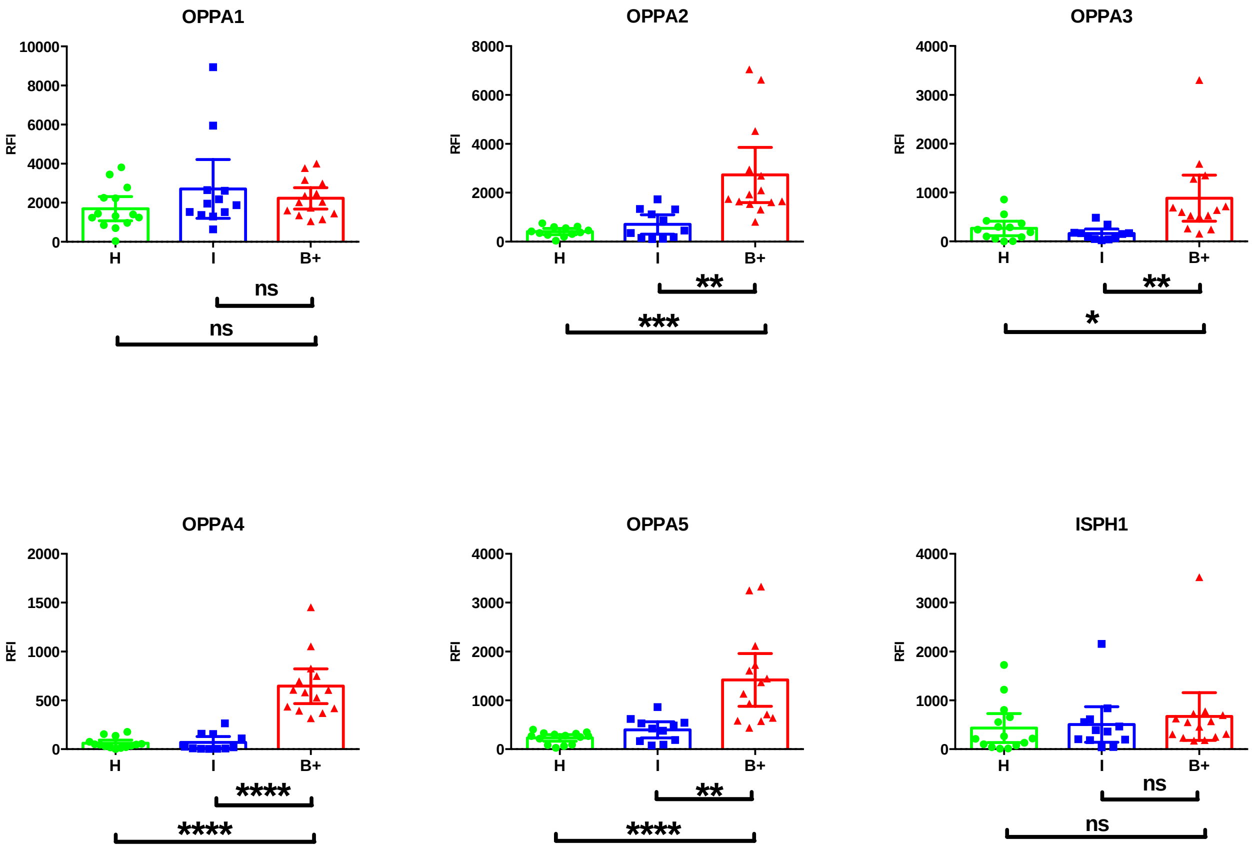


Figure S3: **Antibody recognition signals subdivided by probe and patient category (part II)** : IgG levels specific for peptides OPPA1, OPPA2, OPPA3, OPPA4, OPPA5 and ISPH1 in healthy controls (H), CF patients negative for BCC and positive for other respiratory infections (I) and BCC positive patients (B+). The mean relative fluorescence intensity (RFI), detected at 70% laser power and photomultiplier and P-values of test significance are reported for each peptide.

ns: not significative . Significative: p<0.05; * = p<0.05; ** = p<0.01; *** = p<0.001; **** = p<0.0001.
